# Case in Which the Vena Cava Inferior Was Obstructed from the Commencement of the Common Iliac Veins, and Its Cavity Obliterated between the Entrance of the Emulgent and Hepatic Veins

**Published:** 1845-01

**Authors:** Thomas Bevill Peacock


*Case in which the Vena Cava Inferior was obstructed from the commencement of the common iliac veins, and its cavity obliterated between the entrance of the emulgent and hepatic veins.* By Thos. Bevill Peacock, M. D.—This was a case of complete obstruction of the inferior cava, from the uterine and common iliac veins to the entrance of those from the liver. The obstruction in the former vessels, and the inferior portion of the cava, was the result of the adherent masses of pale lymph, while above, the vessel was converted into a ligamentous cord. The right kidney was in an advanced stage of granular degeneration, the left completely atrophied; the liver was also of small size. The author considered the disease of the vein to have been wholly unconnected with the death of the patient, and ascribed the general dropsy under which she laboured during the last period of her life, to the condition of the kidney and liver. The circulation had been maintained by means of the branches of the vena azygos. The author was of opinion that the adhesions of the uterus to the adjacent organs, and the appearances of the veins, as exhibited in the preparations shown to the Society, were conclusive of the dependence of the obstruction in the vein on inflammation of the vascular tunics.

Mr. Stanley pointed out the features of interest in Dr. Peacock's paper ; viz., the obliteration of the inferior vena cava, and the substitution of a collateral circulation by means of a great enlargement in the vena azygos, without any perceptible increase in size of the superficial abdominal veins.

Dr. Budd remarked that although we were well acquainted with the effects of adhesive phlebitis when occurring in the veins of the extremities, we had not sufficiently attended to the influence of this disease in producing atrophy of the glandular organs. The paper before the Society seemed to him to bear on this subject. He (Dr. Budd) had seen instances in which both the liver and kidney had become atrophied to a great extent from the presence of adhesive phlebitis in the portal and renal veins. He had seen the liver so atrophied as to be indented with deep fissures, and on examination it was found that the branches of the portal veins supplying the atrophied parts were completely obliterated, as the result of phlebitis. He had seen the same effects in the kidney from a like cause.

Mr. Stanley referred to two cases of adhesive phlebitis, which occurred in St. Bartholomew's Hospital; in one, the popliteal vein, in the other, the veins of the arm, were affected with phlebitis, as was evidenced by all the signs and symptoms of that disease; and yet after a time the circulation in the affected veins was completely restored. He inquired if any member present had seen similar cases?

Dr. J. C. B. Williams considered that the pathology of the veins was not sufficiently understood. He took occasion to refer with commendation to Mr. Paget's paper, published some time since, on this branch of pathology, and related a case in illustration. It was an instance in which disease of the veins existed at the same time with disease of the lung. The physical signs were those of pleuro-pneumonia affecting two-thirds of one lung. The symptoms, however, were too severe to be explained simply by this condition, for not only was there extreme difficulty and obstruction in the breathing, but the countenance was remarkably livid, and the œdema of lower extremities rapidly supervened. The patient died, and on examination two lobes of the right lung were in a state of complete carnification, and on being divided by the knife were found to be very red in the interior. The pulmonary veins were much enlarged, and were found to be obstructed by the adherence of large clots to the lining membrane. The vena cava ascendens, and other important veins in the body, were affected in a similar manner, and yet there was no evidence whatever of any recent inflammatory action; on the contrary, the lining membrane was paler and softer than usual, and contained patches of atheromatous deposit. This fact, taken in connection with others of a somewhat similar kind, had led him to consider that many cases supposed to be phlebitis were not so in reality, but that the symptoms presented to us depended on an adherence of the fibrine of the blood, or of coagula, to the coats of vessels, which by the influence of previous disease had become roughened. If we reasoned by analogy, there was good ground to suppose the doctrine correct, for it was well known that the lining membrane of arteries became altered by old age, or some other cause, totally independent of inflammation.

Mr. Fergusson had met with two cases of obstruction in the venæ cavæ, one of the superior, the other of the inferior vein. The first case occurred in a subject in the dissecting-room, and in this instance the collateral circulation had been carried on by the vena azygos greatly enlarged, assisted by the branches which communicate with it. This case bore out the description given by Dr. Peacock in his case, as to the influence of the azygos vein in carrying on the circulation. In the second of these cases, the patient had died three weeks after giving birth to a child, and although she was attended by observant and skilful practitioners, no idea had been entertained by them of the true nature of the case; neither, indeed, were there any symptoms to guide them to an accurate diagnosis. On examination after death, nothing at first was discovered to explain the fatal result. The parts in the neighbourhood of the spine were subsequently examined, and it was then found that the inferior cava was completely blocked up with lymph; it also contained pus in its interior, and there was pus also on its outer surface.

With respect to Mr. Stanley's inquiry, the speaker referred to cases in which varicose veins of the lower extremities had been attempted to be obliterated by the passage of needles under, and string over them. In many cases there was every indication of inflammation having been established; yet, after the expiration of ten, twenty, or forty days, the blood commenced to flow again through these vessels. Cases of the kind were not uncommon, and had probably been seen by many present. These cases had led him to believe, contrary to the common opinion, that adhesive inflammation of the lining membrane of veins was by no means an usual occurrence, nor easily set up.

Dr. Peacock did not agree with Dr. Budd, with respect to the influence of adhesive inflammation on the atrophy of the glandular organs. In his own case he felt convinced that the glandular disease had preceded that in the veins. Besides, the liver in this instance was atrophied, whilst the portal venous system was healthy. The atrophy of the kidney of one side depended on granular degeneration, and the enlargement of the other had its origin in the same disease in a less advanced stage. He considered, in his case, that there were unequivocal proofs that the morbid appearances in the veins were the result of adhesive phlebitis.

Mr. Snow referred the origin of the adhesive phlebitis in Dr. Peacock's case to inflammation of the uterus, in which the veins of that organ had become involved, the disease consequently spreading to the larger vessels. The state of the uterus and its morbid adhesions proved that inflammation had existed in it at some previous time. Mr. Fergusson's last case illustrated the same point.—*Lon. Med. Gaz.*

